# Oral Health Service Delivery in Southeast Asia: A Scoping Review of Access, Delivery, and Barriers

**DOI:** 10.1155/ijod/4906244

**Published:** 2026-10-16

**Authors:** Joseph Macadaeg Acosta, Win Myat Phyo, Rattapong Owasitth, Isi Susanti, Melika Gholamrezaei, Ying Zhou, Silmy Marintan, Pagaporn Pantuwadee Pisarnturakit, Chidsanu Changsiripun, Nipaporn Urwannachotima

**Affiliations:** ^1^ International Graduate Program in Dental Public Health Department of Community Dentistry Faculty of Dentistry Chulalongkorn University Bangkok Thailand chula.ac.th; ^2^ International Graduate Program in Oral Biology Faculty of Dentistry Chulalongkorn University Bangkok Thailand chula.ac.th; ^3^ Department of Community Dentistry Faculty of Dentistry Chulalongkorn University Bangkok Thailand chula.ac.th; ^4^ Department of Orthodontics Faculty of Dentistry Chulalongkorn University Bangkok Thailand chula.ac.th

**Keywords:** access to care, dental care, health equity, oral health services, scoping review, service delivery, Southeast Asia

## Abstract

**Background:**

Oral health is essential for overall well‐being, yet significant disparities in access to oral health services persist in Southeast Asia (SEA). This scoping review examines oral healthcare levels, service delivery models, quality of services, and barriers to accessing oral healthcare services in the region and to identify trends in the current literature on these factors.

**Methods:**

Studies on oral health services in SEA were systematically retrieved from PubMed, Scopus, and Embase, without restrictions on population, language, or publication date, from database inception through July 14, 2024. Study selection and data extraction were conducted independently and in duplicate by at least two reviewers.

**Results:**

Of the 2660 records identified, 52 studies were included after the removal of duplicates, title/abstract screening, and full‐text assessment. These studies covered nine countries, with Thailand, Malaysia, Singapore, and Indonesia most represented. Among the key domains of oral health service delivery reported, accessibility (48.1%), comprehensiveness (32.7%), and coverage (30.8%) emerged as critical factors shaping oral health outcomes. Primary care services were the most frequently mentioned interventions. However, 80.8% of studies identified significant barriers to service delivery, including financial constraints, limited oral health literacy, and geographic challenges—disparities that disproportionately affect low‐income and underserved populations.

**Conclusion:**

This review highlights disparities in oral health services across SEA, with barriers disproportionately affecting low‐income communities. The findings underscore the urgent need to improve accessibility and quality while promoting greater research efforts to inform evidence‐based strategies for oral health equity in the region.

## 1. Introduction

Oral health is a complex aspect of well‐being that encompasses the ability to speak, smile, perceive scents, taste, feel, chew, and swallow [1]. It significantly impacts the physical and mental and exists on a spectrum influenced by individual and societal values and attitudes of individuals and society. Essential attributes for a good quality of life include the physiological, social, and psychological aspects of oral health, which are all influenced by a person’s experiences, perceptions, expectations, and adaptability [1]. According to the Global Oral Health Status Report (2022) from the World Health Organization (WHO), oral diseases represent a major public health issue worldwide [2] and have remained some of the most prevalent health conditions since 1990 [3, 4]. The total estimated number of oral disease cases globally exceeds that of the five major noncommunicable diseases (NCDs)—mental disorders, cardiovascular diseases, diabetes, chronic respiratory diseases, and cancer‐by ~1 billion cases [2].

The WHO defines a health system as comprising all efforts aimed at enhancing, restoring, or sustaining health. This system includes individuals, organizations, and resources structured under established policies to improve population well‐being. Additionally, it aims to meet people’s legitimate health expectations while safeguarding them from financial burdens associated with illness through various health initiatives [5]. To strengthen health systems, the WHO has developed a framework outlining six key components: (i) service delivery, (ii) health workforce, (iii) information, (iv) medical products, vaccines, and technologies, (v) financing, and (vi) leadership and governance [6]. Effective service delivery is crucial for shaping overall population health. The organization and specifics of healthcare services vary significantly between countries [7].

In high‐income countries, oral healthcare systems increasingly focus on treatment and advanced technology, leading to a cycle of interventions that fail to address the root causes of diseases and do not adequately serve large segments of the population. In middle‐income countries, while oral diseases are widespread, oral health care services are often underdeveloped and generally unaffordable for most people [8, 9]. The situation is even more challenging in low‐income countries, where the prevalence of oral diseases is rising. Limited resources and competing health priorities result in very restricted investment in oral health care, making dental services an expensive privilege for the wealthy. Consequently, most people, particularly those in rural areas, cannot access necessary dental treatments [8]. As a result, access to sufficient, high‐quality, and affordable oral health services remains a significant barrier to achieving optimal oral health [10].

Southeast Asia (SEA), as classified by the United Nations (UN), consists of 11 countries: Brunei Darussalam, Cambodia, Indonesia, Lao People’s Democratic Republic (Lao PDR), Malaysia, Myanmar, the Philippines, Singapore, Thailand, Timor‐Leste, and Vietnam [11]. Across both developed and developing nations, several significant barriers impede access to and utilization of oral healthcare services [12]. These challenges include limited oral health literacy, financial constraints, an uneven distribution of dental professionals, and a low prioritization of oral health within healthcare systems. Additionally, cultural norms and beliefs that do not promote healthy behaviors, inconsistent availability of preventive and routine care, inadequate health or dental insurance coverage, insufficient political commitment to integrating oral health into essential healthcare services, and various socioeconomic determinants further contribute to these barriers [10, 12, 13].

Despite the global oral health importance, at present, there is limited evidence‐based research from SEA countries regarding the extent of disparities in access to oral healthcare services. Moreover, there is a lack of comprehensive reviews on the state of oral healthcare services in the SEA. Scoping reviews provide a broad overview, allowing for the exploration of key themes, identification of research gaps, and synthesis of diverse sources of evidence. This scoping review aims to examine the literature on oral health services, focusing on the levels of care, service delivery models, quality of services, and barriers faced by populations in SEA countries. By mapping the existing evidence, this review will provide a foundational understanding of the current state of oral health services in the region, highlight critical gaps in service delivery and access, and inform future research and policy directions to enhance oral health utilization and equity of oral health services.

## 2. Methods

### 2.1. Study Design

This scoping review was conducted in accordance with the Joanna Briggs Institute (JBI) methodology for scoping reviews [14]. The review protocol was registered and published in the Open Science Framework (OSF) (https://osf.io/epj4h/) [15]. Reporting of the review adhered to the Preferred Reporting Items for Systematic Reviews and Meta‐Analyses Extension for Scoping Reviews (PRISMA‐ScR) guidelines [16]. The review was guided by the following research questions: (1) what oral health services are provided within oral health care systems across SEA countries? (2) What are the levels of care, service delivery models, quality dimensions, and barriers to accessing oral health services in SEA?

### 2.2. Eligibility Criteria

Eligibility criteria were developed using the population‐concept‐context (PCC) framework recommended for scoping reviews [17]. Studies were eligible for inclusion if they: (i) reported original empirical research related to oral health service delivery systems; (ii) were conducted in any health care or community setting within SEA countries; and (iii) involved populations of any age group and sex. Eligible study designs included observational studies, qualitative studies, mixed‐methods studies, program evaluations, and policy evaluations. No restrictions were applied regarding the publication year or language.

Studies were excluded if they were systematic reviews, scoping reviews, editorials, commentaries, or conference abstracts, or did not report findings relevant to oral health service delivery. The geographical scope followed the UN classification of SEA, comprising Brunei Darussalam, Cambodia, Indonesia, Lao PDR, Malaysia, Myanmar, the Philippines, Singapore, Thailand, Timor‐Leste, and Vietnam [11].

### 2.3. Search Strategy

A comprehensive literature search was conducted from database inception to July 14, 2024, using three electronic databases: MEDLINE (via PubMed), Scopus, and Embase. To maximize coverage and identify relevant unpublished or nonindexed evidence, gray literature sources were also searched, including Google Scholar, reports from the WHO, Global Burden of Disease (GBD) initiatives, and government publications.

The search strategy combined controlled vocabulary and free‐text terms related to oral health, dental services, healthcare delivery, access to care, and SEA. Boolean operators (“AND” and “OR”) were used to combine the search terms. No restrictions were applied regarding language, population, or publication date. The core search strategy included the following terms: ((oral health) OR (dental health)) AND ((“services delivery system”) OR (“health care service”) OR (“dental services”) OR (“Access to care”) OR (barriers) OR (“health facilities”) OR (hospital) OR (“health center”) OR (“primary care”)) AND ((“Southeast Asia”) OR (SEA) OR (Thailand) OR (Vietnam) OR (Malaysia) OR (Indonesia) OR (Philippines) OR (Singapore) OR (Myanmar) OR (Cambodia) OR (Laos) OR (Brunei) OR (“East Timor”)). The complete search strategies for all databases are provided in Supporting File 1.

### 2.4. Study Selection and Data Extraction

All identified records were imported into Covidence systematic review software (Veritas Health Innovation, Melbourne, Australia), where duplicate records were removed. Prior to the screening, a pilot exercise was conducted to ensure consistency among reviewers. Two reviewers (Nipaporn Urwannachotima and Win Myat Phyo) independently screened the title and abstract against the eligibility criteria. Any disagreements were resolved by consultation with a third author (Pagaporn Pantuwadee Pisarnturakit). Potentially relevant studies subsequently underwent full‐text assessment by four reviewers (Joseph Macadaeg Acosta, Melika Gholamrezaei, Isi Susanti, and Silmy Marintan). Disagreements were resolved through discussion and consensus with other authors.

A standardized data extraction form was developed and pilot‐tested using ~5% of the included studies. Following refinement of the extraction template, studies were independently extracted in pairs (Win Myat Phyo and Rattapong Owasitth, Joseph Macadaeg Acosta and Melika Gholamrezaei, Isi Susanti and Silmy Marintan, and Nipaporn Urwannachotima and Ying Zhou). Any discrepancies were discussed during regular review meetings and resolved by consensus.

No language restrictions were imposed during the search process, although the search terms were in English. All the titles and abstracts identified from the databases were in English, even when the corresponding full‐text articles were published in other languages. Therefore, title and abstract screening did not require translation or any special handling of non‐English records. As no non‐English‐language articles progressed to full‐text assessment, translation and data extraction of non‐English full‐text articles were not required. All studies included in the final review were published in English.

The extracted data included (i) study characteristics (author, publication year, country, objectives, population, study design, and data collection methods); (ii) oral health service characteristics (type of service, provider, and healthcare setting); and (iii) service delivery domains, including comprehensiveness, accessibility, coverage, continuity, effectiveness, safety, patient‐centeredness, timeliness, patient satisfaction, and reported barriers or challenges based on the WHO service delivery monitoring framework [7].

Each included study was reviewed independently to identify all relevant findings. Studies were allowed to contribute to more than one domain if they reported findings relevant to multiple aspects of oral health service delivery. Findings were assigned to the relevant domains, and any discrepancies in categorization were resolved through discussion among the review team until consensus was reached.

### 2.5. Data Synthesis and Presentation of the Results

The extracted findings were synthesized descriptively using a deductive, framework‐guided approach. The WHO framework on the key characteristics of good service delivery [7] provided the conceptual basis for organizing the findings, and the findings were synthesized into six domains: comprehensiveness, accessibility, coverage, continuity of care, quality of care, and barriers to oral health services. Operational definitions were used to guide the categorization of findings. Comprehensiveness referred to the range of oral health services provided in relation to the needs of the target population. Accessibility referred to the extent to which oral health services could be reached and utilized by the target population. Coverage referred to the extent to which the defined target population was reached by or received relevant oral health services. Continuity of care refers to the provision and coordination of care across services, levels of care, or over time. Quality of care encompassed findings related to effectiveness, safety, patient‐centeredness, timeliness, and patient satisfaction. Barriers to oral health services referred to factors reported to hinder access to, utilization of, or delivery of oral health services.

The synthesized findings were presented using narrative summaries, tabulations, and data visualizations. Relevant information was systematically organized into tables to provide a structured summary of study characteristics, oral healthcare services, and findings across the service delivery domains. To analyze trends in reported healthcare barriers, bubble maps were created to visualize their distribution over time, along with an additional visualization illustrating the geographic distribution of reported healthcare barriers across various countries. All visualizations were generated using R Statistical Software (Version 4.5.0; R Core Team, 2025) employing the ggplot2 package.

## 3. Results

The selection process of eligible articles is presented in Figure 1 conforming with the PRISMA 2020 flow diagram [18]. The literature search identified a total of 2660 records, including 2445 records from electronic databases (PubMed, *n* = 1286; Scopus, *n* = 1134; and Embase, *n* = 25) and 215 records from other sources, comprising hand‐searching (*n* = 15) and gray literature (*n* = 200). While none of the 215 records identified through other methods met the eligibility criteria, 1999 records identified through database searches remained for title and abstract screening after 446 duplicate records were removed. Of these, 1882 records were excluded, as they did not meet the inclusion criteria. A total of 117 full‐text reports were sought for retrieval, of which 13 reports could not be retrieved. Consequently, 104 full‐text articles were assessed for eligibility. Following full‐text review, 52 articles were excluded, including 18 due to an irrelevant study design and 34 because they did not report outcomes relevant to oral health service delivery. Ultimately, 52 studies met the eligibility criteria and were included in the scoping review (Figure 1).

**Figure 1:**
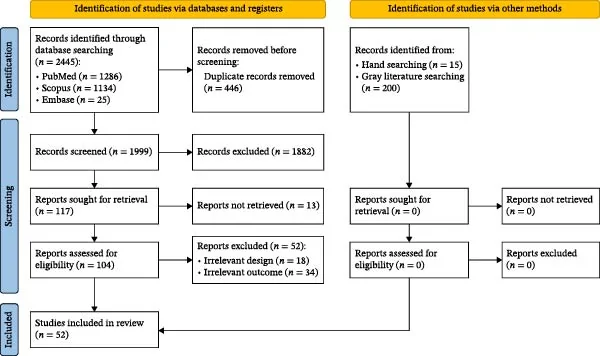
PRISMA flow diagram of the study selection process.

### 3.1. Characteristics of the Included Studies

Among the 52 included studies, nine SEA countries were represented. The most frequently studied settings were Thailand (*n* = 15, 28.8%), Malaysia (*n* = 12, 23.1%), Singapore (*n* = 10, 19.2%), and Indonesia (*n* = 8, 15.4%). Two studies conducted multicountry comparisons within SEA, capturing Brunei Darussalam and Myanmar only in comparative analyses. No included studies specifically focused on Lao PDR or Timor‐Leste as single‐country settings. Publication years ranged from 1998 to 2024 (median 2019, IQR 2010–2022). Sample sizes varied from *n* = 11 to a nationally representative survey record (*n* = 24,952,453) (median 349, IQR 78–799; 41 studies reported a numeric sample size). Most were cross‐sectional (51.9%), with additional qualitative, mixed‐methods, and longitudinal designs. Common data collection approaches were questionnaires/surveys (56.8%), interviews and focus group discussion, clinical examinations (11.5%), and records review (5.8%). All the articles were published in English. Details on the study characteristics are presented in Table 1.

**Table 1:** Characteristic of the included study.

Country	Author, year	Aim of study	Study design	Participant (*n*)	Level of health care
Cambodia	Todd and Durward, 1994 [19]	To describe the current level of utilization of dental services in Cambodia in Phnom Penh	Cross‐sectional study	Adult, elderly (548)	Secondary care, tertiary care
	Chher et al., 2009 [20]	To improve cross‐infection control procedures through the provision of necessary equipment, supplies and training	Nonrandomized experimental study	Dental nurse (n/a)	Primary health care
	Turton et al., 2021 [ 21]	To critically evaluate an early childhood caries intervention performed by nondental primary healthcare providers, considering clinical outcomes, acceptability and stakeholder perceptions	Mixed method	Children, parent, stakeholder (n/a)	Community‐based service
Indonesia	Pasiga, 2018 [22]	To know the behavior of dental health care for Indonesian people	Cross‐sectional study	Adult (180)	—
	Akbar et al., 2019 [23]	To examine the relationship between access to dental health services to dental health conditions in adult communities in urban areas Indonesia	Pathfinder survey	Children, adult, elderly (420)	Primary health care
	Dewanto et al., 2020 [24]	To describe the characteristics of dental services in rural, suburban, and urban areas in the Special Region of Yogyakarta, Indonesia, following the implementation of national health insurance	Cross‐sectional study	Children, adult, elderly (26,554)	Primary health care, secondary care
	Sariyem et al., 2020 [25]	To develop models of dental nursing care in children with deaf and speech impaired	Research and development	Special needs, deaf/speech impaired children, parents/caregivers, teachers (20)	Community‐based service
Indonesia	Akbar et al., 2022 [26]	To conduct a survey on the relationship of sociodemographic characteristics of urban and rural areas with the accessibility of OHS in Pinrang districts, South Sulawesi	Qualitative research	Adult (298)	Community‐based service, primary health care
	Budi et al., 2023 [27]	To investigate descriptive statistics of dental practice during the COVID‐19 pandemic	Pilot study	Dentist (102)	Primary health care
	Khairinisa et al., 2024 [28]	To investigate barriers to providing preventive dental care for preschool children, as perceived by Indonesian general dentists in a clinical setting	Cross‐sectional study	Dentist (362)	—
	Ramphoma et al., 2024 [29]	To explore challenges and proposing context‐specific solutions aligned with the WFPHA maternal oral health framework and its Global Maternal and Child Oral Health Initiative	Case study	Dental health professional (n/a)	Community‐based service, primary health care
Malaysia	Jaafar and Razak, 1988 [30]	To identify variations in utilization among adults in the three major ethnic groups in Malaysia	Retrospective study	Adult, elderly (500)	Primary health care, secondary care, tertiary care
	Jaafar et al., 1992 [31]	To investigate the delay in the utilization of government dental services in Malaysia	Qualitative research	Adult, elderly (555)	Primary health care, secondary care, tertiary care
	Chu and Raja Latifah, 2001 [32]	To investigate the sociodemographic profiles of patients attending public and private dental clinics and the types of treatment received	Cross‐sectional study	Adult (454)	Primary health care
	Othman and Razak, 2010 [33]	To assess the satisfaction with school dental service provided via mobile dental squads in Selangor, Malaysia	Cross‐sectional study	Children (607)	Community‐based service
Malaysia	Saddki et al., 2010 [34]	To determine factors associated with dental visit and to describe barriers to utilization of oral health care services among antenatal mothers attending the obstetric and gynecology specialist clinic in Hospital Universiti Sains Malaysia	Cross‐sectional study	Adult (124)	Community‐based service, primary health care
	Bakar et al., 2019 [35]	To assess equity in healthcare utilization by household SES over time using five series of survey data of the National Health Morbidity Survey	Cross‐sectional study	Household (n/a)	—
	Md Bohari et al., 2019 [36]	To analyze the distribution of public and private dental clinics in relation to the Malaysian population	Cross‐sectional study	General population (24,952,453)	—
	Blebil et al., 2020 [37]	To assess the community pharmacists attitudes, beliefs, and practices toward oral health in the Malaysian setting	Cross‐sectional study	Pharmacist (206)	Primary health care
	Nurdin and Yusof, 2020 [38]	To explore the facilitators and barriers to the implementation of the POHP using the perspectives of dental therapists in Selangor state, Malaysia	Qualitative research	Adults (114)	Community‐based service
	BenGhasheer and Saub, 2022 [39]	To assess parental oral health KAP, perceptions, and explore the barriers to dental care utilization among Libyan parents living in Malaysia	Cross‐sectional study	Immigrant, Libyan parents (381)	Primary health care
	Ali et al., 2023 [40]	To assess the spatial accessibility of public oral healthcare facilities with the SDS mobile team among Selangor primary and secondary schoolchildren in terms of traveling distance and time	Cross‐sectional study	Children (524,876 primary school students and 340,873 secondary school students)	Primary health care, secondary care
Malaysia	Mohd Khairunjauhari et al., 2023 [41]	To explore the oral health beliefs, perceptions, and oral health service utilization behavior among Orang Asli in the district of Bera, Pahang, Malaysia	Qualitative research	Adult, elderly (19 indigenous)	—
Philippines	Ofilada and Jimeno, 2013 [42]	To determine the factors that prevented type 1 diabetic patients from seeking dental treatment in Metro Manila, Philippines	Cross‐sectional study	Children, adult (78)	Community‐based service, primary health care
Singapore	Lo et al., 1991 [43]	This study determined the dental service utilization rate among disabled children and factors influencing their utilization of services	Cross‐sectional study	Children (322)	Community‐based service, primary health care, secondary care
	Soh, 1991 [44]	To determine the adequacy of arrangements for dental care in long‐term care facilities in Singapore	Cross‐sectional study	Chef administrators of elderly homes (68)	Community‐based service, primary health care
	Lim, 2019 [45]	To provide a demographic analysis of patients with special care needs and to improve the oral health of seniors or adults with disabilities facing barriers to care	Retrospective descriptive study	Adult, elderly (55 special needs)	Primary health care, secondary care, tertiary care
	Mittal et al., 2019 [46]	To understand the factors affecting dental care utilization among older Singaporeans who were eligible for CHAS or PG subsidies	Qualitative research	Elderly (25)	Community‐based service
	Teo et al., 2020 [47]	To report on the oral health status, disability‐related barriers, and assess how these barriers limit care pathways	Cross‐sectional study	Special needs (29)	Community‐based service, primary health care
Singapore	Ho et al., 2023 [48]	To evaluate the perceptions of nursing homes staff and oral health therapists regarding the barriers and enablers toward oral care provision for elderly nursing homes residents, and their perceptions toward interprofessional collaboration through the utilization of oral health therapists in nursing homes	Cross‐sectional study	Oral health therapist, nursing home staff (349)	—
	Junnarkar et al., 2023 [49]	To determine the barriers to dental care for autistic children in Singapore through the experiences and opinions of their parents	Qualitative research	Parents of ASD children 3‐12 years (23)	Community‐based service, primary health care, secondary care, tertiary care
	Ho et al., 2024 [50]	To evaluate the perceptions of oral health therapists regarding the barriers towards oral care provision for the elderly residents in nursing homes	Qualitative research	Oral health therapist (11)	—
	Koh et al., 2024 [51]	To identify dentists’ perspectives on structural and system barriers that hinder oral healthcare provision to seniors in NHs in Singapore to understand the consequences of these barriers	Qualitative research	Dentist (19)	—
	Tan et al., 2024 [52]	To assess the influence of income and financial subsidies on the utilization of dental care services among persons with intellectual and/or developmental disabilities in Singapore	Cross‐sectional study	Special needs and caregivers (591)	—
Thailand	Benjakul and Chuenarrom, 2000 [53]	To assess the actual need and patient demand for dental care at the University Dental Hospital, PSU, to improve service planning and resource allocation	Quantitative study	Children, adult, elderly, special needs (4175)	Community‐based service, primary health care, secondary care, tertiary care
	Somkotra and Detsomboonrat, 2009 [54]	To assess the socioeconomic‐related inequality and horizontal inequity in oral healthcare utilization among Thai adults after the universal coverage policy	Cross‐sectional study	Adult (32,748)	—
	Somkotra and Vachirarojpisan, 2009 [55]	To quantify the socioeconomic inequality in dental care utilization among Thai children and examine the characteristics of such utilization after the country achieved universal coverage	Cross‐sectional study	Children (15,736)	Community‐based service, primary health care, secondary care, tertiary care
	Tianviwat et al., 2009 [56]	To identify the optimal levels and mix of basic dental services under two different dental settings (hospital‐based and mobile dental clinics) with specified resource constraints	Modeling study—linear programming	Children and parent pair (210)	Primary health care
	Tianviwat et al., 2009 [57]	To compare the performance of hospital clinics with and without adjunct mobile services for the delivery of secondary prevention for caries in Thai schoolchildren	Cohort study	Children (711)	Secondary care
	Somkotra, 2013 [58]	To assess socioeconomic‐related inequality in oral healthcare utilization among older Thais	Cross‐sectional study	Elderly (20,353)	—
Thailand	Somkotra, 2013 [59]	To assess the socioeconomic‐related inequality in dental care utilization among Thai elderly after the country achieved universal coverage and to determine the associated factors	Cross‐sectional study	Elderly (10,096)	Primary health care, secondary care, tertiary care
	Chongthanavanit and Kheokao, 2018 [60]	To study factors influencing foreign tourists decisions to use dental services in Thailand	Cross‐sectional study	Tourist (270)	Primary health care, secondary care, tertiary care
	Sermsuti‐Anuwat and Pongpanich, 2018 [61]	To explore the perspectives and experiences of Thai wheelchair users regarding barriers of access to dental services and report potential solutions in terms of customer satisfaction	Mixed method	Special needs (156)	—
	Limpuangthip et al., 2019 [62]	To assess the determinants associated with inequalities in denture service utilization among older Thai people using the Andersen behavioral model	Cross‐sectional study	Elderly (38,695)	Tertiary care
	Harirugsakul et al., 2020 [63]	To determine the associations between social backgrounds, oral behaviors and dental service utilization among Thai older adults	Cross‐sectional study	Elderly (4130)	—
	Thitasomakul et al., 2020 [64]	To assess the cost effectiveness of a hospital‐based dental clinic versus a mobile dental clinic for a school dental sealant program in Southern Thailand	Economic evaluation	Children (2744)	Primary health care
	Chongthawonsatid et al., 2021 [65]	To identify unmet healthcare needs, including oral care, and examine the factors influencing access to healthcare services in Thailand	Health survey	Children, adult, elderly	—
Thailand	Somboonchan et al., 2022 [66]	To determine the health care supplies needed for the dental health team conducting home visits to dependent older adults in Phitsanulok Province through a needs assessment survey, and to understand the oral health services and supplies requirements of dependent older adults	Cross‐sectional study	Dentist, dental therapist, dental public health practitioner (176)	Community‐based service
	Chaianant et al., 2023 [67]	To evaluate socioeconomic inequalities in dental service utilization before and after the implementation of the Universal Coverage Scheme in Thailand	National survey	Adult (14,050)	—
Vietnam	Dao et al., 2015 [68]	To identify the prevalence and severity of adult oral health problems presenting at CHCs and to examine the use of oral health services and self‐medication in response to these problems	Cross‐sectional study	Adult (799)	—
Brunei; Cambodia; Malaysia; Myanmar; Philippines; Indonesia; Singapore; Thailand; Vietnam	Petersen and Phantumvanit, 2012 [69]	To discuss current information on fluoride and dental caries, as well as to try identifying barriers and opportunities that countries of Asia may have for implementing such programs	—	—	Community‐based service, primary health care
Cambodia; Malaysia; Indonesia; Thailand	Ng et al., 2022 [70]	To identify barriers to early detection and management of oral cancer in the Asia Pacific region	Mixed method	Adult (14)	—

### 3.2. Oral Health Service Delivery

Service delivery is a fundamental component of the health system and serves as the immediate outcome of investments made in other areas, such as the workforce, financing, information, medicines and technologies, and governance. Oral health service delivery was assessed using an adapted WHO service delivery framework encompassing comprehensiveness, accessibility, coverage, continuity, quality, and barriers to care [7]. The data extracted from included studies by country are shown in Figure 2, with a detailed extraction for all studies available in Table S1. Many studies reported multiple aspects of oral health service delivery; individual studies could be represented in more than one domain. Therefore, the frequencies reported for each domain are not mutually exclusive. Across domains, the most frequently reported were accessibility (48.1%), comprehensiveness (32.7%), and coverage (30.8%), emphasizing their central role in influencing oral health outcomes. Notably, a large proportion of studies (80.8%) identified barriers or challenges to service delivery, highlighting persistent limitations within oral health systems across the region. Among the included studies, preventive and promotive services were more frequently reported than rehabilitative and specialized care. Although service delivery models varied across settings, several studies have reported challenges related to affordability, workforce availability, and geographic access.

**Figure 2:**
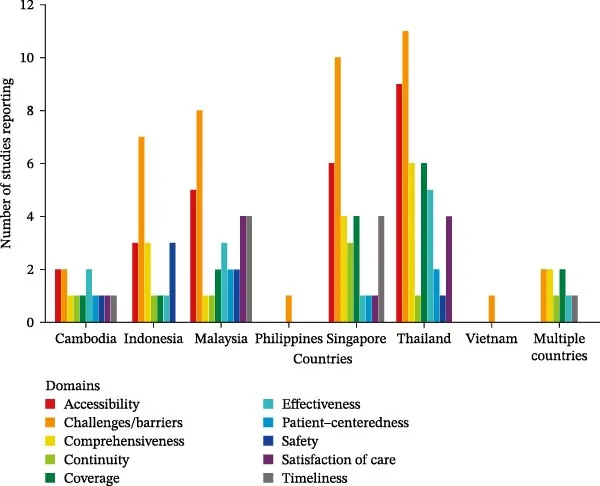
Oral health service delivery domains by country.

#### 3.2.1. Comprehensiveness

Analyzed through the lens of universal health coverage (UHC) and health systems strengthening, the range of oral health services reported across studies suggests areas for attention, particularly in service comprehensiveness, financial protection, and equity. Preventive and health promotion interventions were most frequently described, particularly school‐ and community‐based programs such as oral health screening, fluoride application, fissure sealants, and education activities (*n* = 22, 42.3%). Basic curative services were also common, including restorative care (*n* = 19, 36.5%) and extractions (*n* = 15, 28.8%).

However, from a UHC perspective, the included studies provided less frequent evidence on more complex clinical services, with periodontal care (19.2%), prosthodontics (17.3%), and endodontics (11.5%) reported less frequently than other service domains. Specialized services—such as referrals (7.7%), orthodontics (5.8%), diagnostic imaging (1.9%), and emergency or teledentistry—were rarely reported, with only one study mentioning teledentistry. The lower frequency of reporting of advanced interventions compared with basic preventive services suggests that the available literature provides less evidence on the delivery of comprehensive oral health services, particularly for more complex conditions. The distribution of service types across countries is illustrated in Figure 3 showing a similar pattern in service composition across the reported country.

**Figure 3:**
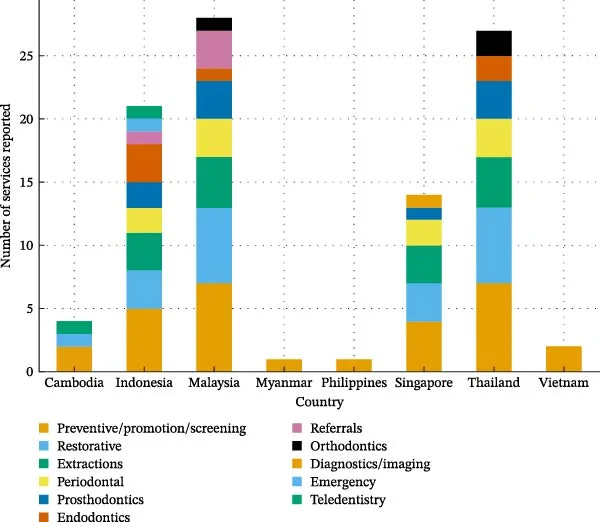
Number of oral health services type reported by country.

Patterns of service provision also demonstrated variability in delivery systems and the workforce. Mixed public–private delivery models were the most frequently cited (32.7%), followed by public systems (28.8%), while a significant proportion of studies did not specify the setting. Several studies have also identified socioeconomic and financial barriers to accessing oral health services. Task‐shifting was reported as a strategy for expanding access in some settings, while workforce distribution was characterized by geographic or organizational concentration in several studies.

Dentists were the principal providers (51.9%), with auxiliary dental personnel such as hygienists, therapists, and nurses supporting delivery in nearly a quarter of studies (23.1%). A few references were made to broader cadres, including community or public health workers, long‐term care staff, traditional practitioners, and pharmacists. The included studies predominantly reported service delivery involving dentists, while the use of mid‐level auxiliary personnel was less frequently documented. This pattern indicates that the available literature provides relatively limited evidence on the role of auxiliary personnel in oral health service delivery. Overall, preventive and basic treatment services were more frequently reported than specialized and rehabilitative care.

#### 3.2.2. Accessibility

Accessibility to oral health services emerged as a recurring theme, with nearly half (48.1%) addressing issues related to access or utilization. The majority of the oral health service was delivered through public hospital dental departments (28.8%), followed by dental clinics (13.5%), school settings (11.5%), long‐term care facilities (7.7%), health centers (5.8%), mobile clinics (3.8%), and community‐based initiatives (3.8%). Approximately half of the studies indicated that services were located near communities through primary care entry points (50.0%), with the next major category being community‐based delivery (30.8%), followed by secondary care (23.1%) and tertiary care (15.4%). Several studies have also reported the integration of services across multiple levels of care. Many included studies reported barriers to access, including financial constraints, geographic inequities, and organizational limitations. Where utilization data were available, reported utilization was generally lower than the benchmarks cited in the respective studies and was associated with socioeconomic and geographic differences in accessibility.

National and subnational analyses from Thailand [54, 55, 59] and Malaysia [36] indicated that while there is universal or broad public coverage, the reported rates of dental visits over a 12‐month period remain low and significantly influenced by income, education, and rural–urban residence. In Singapore, only 6% of long‐term care facilities provide in‐house dental clinics [44]. Evidence suggested that targeted subsidies for treatment costs and transportation support could improve utilization among priority groups, such as the elderly and patients requiring special care [47]. In Indonesia, urban populations have better access to facilities and infrastructure, whereas rural residents face significant barriers, including distance, travel time, and out‐of‐pocket costs [23]. The included studies frequently reported barriers to accessibility, including geographic location, transportation availability, educational attainment, and socioeconomic status. Accessibility challenges were also reported in some settings with broad public coverage or subsidized dental services. Across settings, the reported evidence indicates that access to oral health services may be influenced not only by the availability of services but also by populations’ ability to physically reach and effectively utilize them.

#### 3.2.3. Coverage

Analyzed through the framework of UHC, approximately one‐third of the included studies (30.8%) reported on coverage or entitlement schemes. Target groups most frequently addressed were children and adolescents (32.7%) and older adults (32.7%), followed by general or unspecified populations (23.1%) and adult patients in clinical settings (17.3%). However, only one study specifically focused on migrants and refugees. The included studies showed greater attention to specific population groups, particularly children and older adults, while vulnerable and transient populations were less frequently represented in the reported evidence.

The coverage of oral health services in the SEA shows substantial heterogeneity across countries, reflecting differences in policy frameworks, financing mechanisms, and service delivery models. Several countries, notably Malaysia and Singapore, have implemented comprehensive population‐based preventive measures such as community‐wide fluoride administration, alongside broader health system initiatives, including cancer surveillance registries in Malaysia and Thailand [69, 70]. Preventive programs for children are also widely reported, such as the Incremental Dental Care Program targeting school‐aged children and nationwide tooth‐brushing programs with high population reach [33]. Thailand’s Universal Coverage Scheme (UCS) has limitations regarding advanced care [54, 61]. Similar limitations are observed in Singapore, where subsidies such as the Community Health Assist Scheme (CHAS Blue) had only marginal effects on utilization among vulnerable populations, including individuals with intellectual and developmental disabilities [46]. In Malaysia, although government dental services are widely available at subsidized fees, disparities persist. For example, Selangor has the lowest chair‐to‐population ratio (1:6000) despite being the most populous state [36].

Coverage is also influenced by the type of insurance, sponsorship, and household income. Evidence consistently shows that higher‐income groups are more likely to utilize dental services, highlighting persistent socioeconomic disparities among the reported countries [32, 46, 52, 54, 55, 61, 67]. Facilities such as long‐term care centers exhibit variable coverage [44], and while mobile dental services have been reported to slightly increase access, the coverage outcomes remain lower compared to those of fixed facilities [56]. However, coverage of rehabilitative and specialized services was less consistently reported. While countries with established public financing mechanisms generally achieved wider population coverage, disparities in service utilization persisted according to income, insurance status, and geographic location.

#### 3.2.4. Continuity of Care

The continuity of care within oral health services in SEA is documented inconsistently, with evidence primarily found in specific national programs and among particular population groups. Notably, Malaysia demonstrates one of the earliest and most enduring examples of lifecycle‐based continuity through its school dental service, first implemented in 1948. This program has successfully provided sustained preventive care throughout childhood and adolescence [33]. Similarly, several countries have reported integrating oral health into existing health platforms, such as incorporating dental care into six scheduled contacts during routine childhood vaccination programs. Several included studies reported integrating oral health activities into existing maternal and child health (MCH) services, suggesting that established primary care platforms may provide opportunities for earlier and more coordinated delivery of oral health prevention [29, 66].

For vulnerable groups, various initiatives have been developed to maintain ongoing access to care. In Thailand, home‐visit models have been recommended for providing oral health care to dependent older adults, underscoring the importance of continuity for populations with limited mobility [66]. In Indonesia, a project in West Kalimantan illustrated how oral health promotion can be integrated and sustained as part of prenatal care, positioning oral health within a broader MCH framework [29]. However, the establishment of formal clinical entry points does not necessarily ensure the continued utilization of oral health services. The included studies also described the referral mechanisms for dental treatment within disability services. Nevertheless, regular utilization remained limited in some settings, with one study reporting that caregivers perceived dental care as unnecessary in the absence of visible or acute oral health problems [43].

Polyclinic systems have supported continuity by allowing patients to see the same dentist over time, even after receiving subsidized care. Nonetheless, challenges remain in ensuring long‐term follow‐up for chronic or complex conditions [46]. The included studies reported limited evidence of formalized referral and follow‐up pathways linking primary and tertiary oral health services. Participants in several studies identified the need for one‐stop centers for conditions such as oral cancer and for online tracking systems to support patient follow‐up [70]. These findings indicate that the available literature has identified opportunities to strengthen referral coordination, information sharing, and continuity of care for patients with complex oral health conditions.

#### 3.2.5. Quality of Care

Quality domains identified in oral health care were effectiveness (25.0%), timeliness (19.2%), safety (13.5%), and patient‐centeredness (11.5%). Variability in reporting reduced comparability between studies.

Evidence from the SEA demonstrates that various oral health interventions have achieved measurable effectiveness. School‐ and community‐based programs, particularly mobile dental clinics, have been recognized as cost‐effective strategies for expanding preventive services among children [33, 43]. Studies indicated significant improvements in knowledge, attitudes, and skills among participants following oral health education interventions [25]. Preventive measures such as sealants, fluoride applications, and early screening have resulted in decreased odds of early childhood caries and improved oral health‐related quality of life [21]. Secondary prevention delivered through both hospital and mobile dental services has shown high effectiveness rates, reaching up to 97% [57]. Optimization studies suggested that reconfiguring the balance between mobile and fixed services could enhance the willingness‐to‐pay outcomes by more than 75% [56]. Collaboration among multidisciplinary teams—including dental professionals, doctors, nurses, teachers, caregivers, and health volunteers—has been effective in improving the delivery of oral health and fostering stronger cooperation from the target population [38, 53, 66].

Adherence to safety standards has been inconsistent. Positive practices included the widespread use of PPE, biosafety protocols, antiseptic gargling, and external suction devices, especially during the COVID‐19 pandemic [27]. Dental home visits often involve multidisciplinary teams, which ensure integrated and safe care for dependent populations [66]. However, risks have also been reported, especially concerning unregulated dental “handymen” operating outside the formal system and insufficient safety standards in some facilities [26]. While most community health centers comply with Ministry of Health regulations regarding equipment and diagnoses, concerns about gaps in regulatory oversight persist [24, 37].

Patient‐centeredness has not been consistently prioritized. Some groups recognize the value of oral health services for improving overall well‐being and express satisfaction with school and community support in facilitating care [28]. Patients tend to prefer professional dentists over traditional providers, indicating a demand for formalized, quality care [19]. Several studies reported that oral health services were not always tailored to the needs of specific populations, including individuals with disabilities, particularly wheelchair users and people with autism spectrum disorder [61]. In addition, one study conducted at a university dental hospital reported that approximately one‐quarter of patients did not receive the treatments they had requested [53].

Timeliness was another recurrently reported dimension of service delivery. Delays in accessing dental care were attributed to long waiting times [34, 36], work commitments, and the perception that dental problems were not urgent [31, 43]. In some public‐sector settings, studies also reported delays related to insurance approval and specialist referral processes, with some patients subsequently seeking care from private providers. Emergency‐oriented patterns of dental care, including tooth extraction and restorative treatment, were reported in some studies [43]. Overall, the included studies predominantly evaluated service effectiveness, while fewer studies examined other dimensions of service quality, such as timeliness, responsiveness, continuity, and patient‐centeredness.

#### 3.2.6. Barriers to Oral Health Services

Barriers to oral health services were reported in 80.8% of the studies. The most common barriers included financial constraints (32.7%), transportation (25.0%), provider shortages and long waiting times (23.1%), and limited oral health literacy (19.2%). As shown in Figure 4, the distribution of these barriers is consistent across various years and countries, particularly in more recent and frequently studied settings.

**Figure 4:**
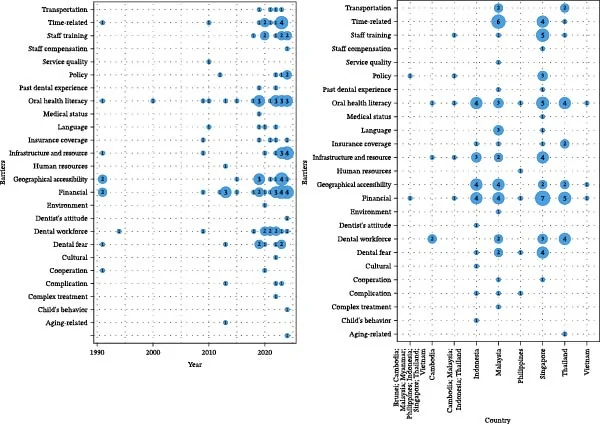
Barriers to oral health care services by year and country.

Financial and indirect costs were frequently reported as barriers to accessing oral health services in the included studies. High treatment costs and limited insurance coverage were reported in several SEA settings, including among low‐income populations and individuals with disabilities [22, 26, 35, 52, 58]. Barriers were also reported in settings where dental services were provided at no or subsidized cost. For example, studies of UHC subsidies and a public denture program in Thailand identified transportation and other indirect costs as factors affecting access [48, 62]. Out‐of‐pocket payment was commonly reported, with some studies describing greater use of private dental services among higher‐income populations and delayed or forgone treatment among individuals with lower socioeconomic status [36, 55, 62]. Several studies also reported that insurance schemes primarily covered basic or primary dental treatments, with more comprehensive services less frequently included [54, 59, 70]. Preventive programs, including fluoride programs and oral cancer screening, were also reported in some studies as having limited funding or implementation [69]. In addition, several studies reported gaps in health information systems and limited availability of national registries, including oral cancer surveillance systems in Cambodia and Indonesia [70].

Several included studies reported challenges in accessing oral health care among rural populations, including limited availability of dental facilities and professionals, long travel distances to obtain care, and transportation‐related barriers [36, 44, 64–66]. The included studies reported a greater concentration of private dental services in urban areas, alongside reported differences in access between rural and urban populations [23, 33, 35]. Although mobile dental clinics have been introduced to extend coverage to underserved areas, their services often focus primarily on tooth extractions and provide limited preventive and promotive care, such as oral health education, fluoride application, and early detection of oral diseases [33, 43, 57]. Several studies reported a greater emphasis on curative services than preventive care in rural settings, with some also reporting unmet oral health needs among rural populations.

Individuals with lower levels of education often perceive oral health as a low priority, leading to a reliance on self‐medication and traditional providers instead of formal dental services [62, 68]. Several misconceptions persist, such as the belief that primary teeth do not require treatment, that dental care is only necessary when symptoms arise, and that visiting a dental clinic poses a risk of cross‐infection [39, 42]. Additionally, fear, anxiety, and negative past experiences with dental discourage timely treatment‐seeking behavior [27, 34, 41, 46].

Several included studies described organizational and service delivery challenges, including short consultation times, long waiting lists, and difficulties in accessing services within public facilities [20, 39]. Patient dissatisfaction and delays in care were also reported in some settings. In addition, limited multidisciplinary coordination was reported in relation to preventive programs and the provision of oral health services for individuals with special needs [64]. In nursing homes and long‐term care settings for dependent older adults, service provision is often constrained by reliance on volunteer dentists, limited training in geriatric dentistry, and the lack of on‐site facilities, equipment, and appropriate remuneration for providers [43, 50]. Workforce‐related challenges continue to constrain oral health service delivery. Several included studies reported shortages and uneven distribution of qualified dental professionals in rural and remote areas [19, 20, 63]. Younger dentists and those in the public sector frequently report heavy workloads and limited institutional support for preventive initiatives. In some settings, these factors were associated with a greater emphasis on curative rather than preventive services [28]. Among people with disabilities, studies reported additional barriers to accessing oral health care, including refusal of services, limited staff training, and insufficient time or resources to address complex treatment needs [61].

## 4. Discussion

This scoping review provides the most comprehensive synthesis to date of oral health service delivery across SEA, examining service organization, accessibility, coverage, quality, and barriers through the WHO service delivery framework [7]. The findings reveal substantial heterogeneity in oral health systems across the region, yet several common patterns emerged. Preventive and promotive services, particularly school‐based oral health programs, fluoride initiatives, and community outreach activities, were widely reported, whereas rehabilitative, prosthodontic, endodontic, and other specialized services were less consistently available. This pattern suggests that although many SEA countries have made progress in expanding basic oral health services, access to comprehensive and continuous care remains uneven. Such disparities reflect broader challenges in balancing preventive and treatment‐oriented approaches within health systems and are consistent with observations from other low‐ and middle‐income countries (LMICs), where oral health services frequently remain concentrated on essential care rather than comprehensive disease management [8, 71]. Accessibility has emerged as one of the most prominent dimensions of service delivery. Across countries, utilization of oral health services was strongly influenced by socioeconomic position, educational attainment, geographic location, and health system factors. Notably, the persistence of low dental attendance rates in countries with established public financing mechanisms, including Thailand and Malaysia, suggests that financial coverage alone is insufficient to ensure equitable utilization.

Quality of care domains were variably addressed, while effectiveness of community and mobile services was reported, timeliness and patient‐centeredness were recurrent weaknesses, consistent with global concerns about dental system responsiveness [72]. Furthermore, vulnerable populations, including migrants, refugees, persons with disabilities, and dependent older adults, were underrepresented in service provision despite experiencing disproportionately high oral health needs [43, 44, 46, 61, 66]. This finding highlights a persistent mismatch between population needs and the design of oral health services across the region.

Differences in oral health service delivery between SEA countries appear to be closely linked to variations in health system organization, financing arrangements, workforce distribution, and public–private sector involvement. Thailand’s UCS has facilitated broad population access to essential dental services [54, 61], while Malaysia has developed an extensive subsidized public dental system complemented by private sector provision [36]. Singapore’s mixed financing model combines targeted subsidies with a substantial private dental market [46], whereas Indonesia continues to face challenges associated with geographic fragmentation, infrastructure limitations, and uneven workforce distribution [23]. Although these systems differ considerably, a common observation across countries is that populations residing in rural and underserved areas continue to experience reduced access to care [23, 36,,41, 45, 63]. These findings suggest that oral health inequalities are not solely a consequence of individual socioeconomic circumstances but are also shaped by the way health systems allocate resources, organize service delivery, and prioritize oral health within broader health agendas.

Barriers to oral health service delivery were identified in more than 80% of studies, led by financial constraints, transportation difficulties, provider shortages, and long waiting times. While universal coverage policies and subsidized public services provide an important foundation for improving oral health equity, the present review demonstrates that financial protection alone cannot eliminate disparities. Indirect costs, including transportation expenses, travel time, lost income, and caregiver burden, remain significant barriers, particularly for rural populations, dependent older adults, and people with disabilities. Moreover, many insurance schemes primarily cover basic dental services while offering limited support for rehabilitative, prosthodontic, or specialist care. Consequently, individuals requiring more complex treatment continue to face substantial financial and organizational barriers. Similar patterns have been observed in other health systems, where gaps between service availability and actual accessibility persist despite formal coverage arrangements [73].

From a health systems strengthening and UHC perspective, the findings suggest that oral health inequities in SEA are embedded within broader system‐level constraints. Workforce maldistribution, limited investment in preventive services, gaps in referral pathways and oral health surveillance systems, and the concentration of private providers in urban centers collectively may contribute to persistent inequalities in service access and utilization. Importantly, countries that demonstrated broader preventive coverage were generally characterized by stronger public financing mechanisms and more established primary healthcare infrastructures. Nevertheless, even in these settings, challenges related to continuity of care, patient‐centeredness, and equitable access remained evident. These findings reinforce the growing recognition that achieving UHC for oral health requires more than expanding treatment coverage; it necessitates coordinated reforms across finance, workforce planning, governance, service integration, and health information systems.

The findings may have implications for oral health policy across SEA and can be considered within the broader global health context, particularly in relation to health equity, UHC, and health systems strengthening [74]. Given the shared challenges identified across the region, including workforce shortages, geographic inequities, and underinvestment in preventive care and surveillance systems, greater regional collaboration may complement national reform efforts. Mechanisms such as knowledge exchange, workforce capacity building, and harmonization of oral health indicators could support more coordinated approaches to addressing common oral health challenges across SEA countries. At the national level, reducing geographic disparities will require strategic redistribution of the oral health workforce through incentive‐based deployment programs, strengthened rural retention policies, and expanded utilization of mid‐level oral health providers, consistent with WHO recommendations on human resources for health [75]. Financing reforms should move beyond coverage of basic emergency and restorative services toward comprehensive essential oral health benefit packages that include preventive, rehabilitative, and selected specialist services while minimizing out‐of‐pocket expenditures. Furthermore, oral health should be more effectively integrated into primary healthcare systems and national NCD strategies, reflecting the growing recognition of oral diseases as a significant public health concern. Sustained investment in preventive interventions, including fluoride programs, school oral health services, and oral cancer screening initiatives, will be critical to reducing the long‐term disease burden and treatment costs. Strengthening oral health information systems and integrating oral health indicators into broader health surveillance platforms are also essential for evidence‐informed planning, monitoring, and accountability [76]. Collectively, these strategies support the transition toward more equitable, integrated, and people‐centered oral healthcare systems, consistent with the objectives of the WHO Global Oral Health Action Plan [77].

Some limitations should be considered when interpreting the findings. Although comprehensive search strategies were employed, the available evidence was identified from only a subset of SEA countries and was heavily concentrated in Thailand, Malaysia, Singapore, and Indonesia. In contrast, little or no eligible evidence was identified from countries such as Lao PDR and Timor‐Leste. This should not be interpreted as indicating the absence of oral health services in countries without eligible studies. It may reflect limited research output, lower publication visibility, or indexing practices rather than actual differences in the oral health system. Consequently, the findings represent the available published evidence rather than the full extent of oral health service delivery across the region. The absence of evidence from certain countries represents an important regional knowledge gap and highlights the need for greater investment in oral health systems research. In addition, although gray literature sources were searched, this review did not aim to provide a comprehensive country‐by‐country analysis of oral health policies. Given the breadth of the review and variability in the availability of policy documents across countries, a dedicated policy analysis is beyond the scope of the present study. Future reviews may benefit from a more detailed synthesis of national oral health policies and service delivery models to complement the findings of this review.

Although quality appraisal was not conducted in accordance with the scoping review methodology, the characteristics of the included evidence should be considered when interpreting the findings. Most studies employed cross‐sectional designs, limiting the causal inference and evaluation of long‐term outcomes. Considerable heterogeneity was observed in study populations, outcome measures, and reporting of service delivery domains, which restricted direct comparison across countries. Furthermore, relatively few studies have evaluated system‐level reforms, financing mechanisms, or longitudinal changes in oral health service delivery.

Future research should move beyond descriptive assessments of service utilization and access toward more comprehensive evaluations of oral health systems. Longitudinal and mixed‐method studies are needed to examine the impact of financing reforms, workforce redistribution strategies, preventive interventions, and primary healthcare integration on oral health outcomes and equity. Greater attention should also be given to underrepresented populations, including migrants, refugees, persons with disabilities, and residents of remote communities. Finally, strengthening research capacity and health systems research in underrepresented SEA countries will be critical for generating context‐specific evidence and ensuring that future regional policies reflect the diverse realities of oral health service delivery across the SEA.

## 5. Conclusion

This review highlights persistent disparities in oral health service delivery across SEA, with barriers disproportionately affecting low‐income and vulnerable populations. Based on the currently available published evidence, oral health service delivery has been described primarily in a subset of SEA countries. Despite progress through school programs, UCS, and preventive initiatives, inequities remain due to underfunded prevention, weak data systems, and treatment‐oriented models. Addressing these gaps will require greater investment in prevention, stronger surveillance, a redistribution of the workforce, and integration of oral health into broader health system reforms. Coordinated regional collaboration and expanded research are essential to improving accessibility, quality, and equity in oral health services across the region. Additional research from underrepresented countries is needed to provide a more comprehensive understanding of the region.

## Author Contributions

**Joseph Macadaeg Acosta and Win Myat Phyo:** conceptualization, methodology, investigation, data curation, writing – original draft, writing – review and editing. **Rattapong Owasitth**: methodology, investigation, data curation, visualization. **Isi Susanti, Melika Gholamrezaei, Ying Zhou, and Silmy Marintan**: methodology, investigation, data curation. **Pagaporn Pantuwadee Pisarnturakit**: methodology, investigation, writing – review and editing. **Chidsanu Changsiripun**: conceptualization, methodology, investigation, data curation, supervision, writing – original draft, writing – review and editing. **Nipaporn Urwannachotima**: conceptualization, methodology, investigation, data curation, visualization, supervision, writing – original draft, writing – review and editing.

## Funding

Chidsanu Changsiripun was supported by the Irish Health Research Board and the HSC Public Health Agency (Grant ESI‐2021‐001) in association with Evidence Synthesis Ireland/Cochrane Ireland.

## Disclosure

All authors have read and approved the final version of the manuscript. Nipaporn Urwannachotima and Chidsanu Changsiripun had full access to all of the data in this study and take complete responsibility for the integrity of the data and the accuracy of the data analysis.

## Ethics Statement

This scoping review used only publicly available published literature and did not involve human participants or identifiable personal data. Therefore, ethical approval and informed consent were not required.

## Conflicts of Interest

The authors declare no conflicts of interest.

## Supporting Information

Additional supporting information can be found online in the Supporting Information section.

## Supporting information


**Supporting Information** Supporting Information file: Supplement 1 provides the complete search strategies for all databases and Table S1 presents the extracted service delivery domains from the included studies.

## Data Availability

All the data used in this scoping review were obtained from published studies. The complete extracted data supporting the findings of this review are available from the corresponding authors upon reasonable request.
