# Supplementary material for: Oral Health Service Delivery in Southeast Asia: A Scoping Review of Access, Delivery, and Barriers
Source: Int J Dent. 2026 Oct 16;2026:4906244. doi: 10.1155/ijod/4906244 (PMC13674008; doi:10.1155/ijod/4906244)
Supplement: Supplementary file 1 [file IJOD-2026-4906244-s001.docx]

#### Supplementary files

# Supplement 1: Search strategy

**PubMed/MEDLINE**

1. (("oral health"[MeSH Terms] OR "oral health"[Title/Abstract] OR "dental health"[Title/Abstract]))
2. AND
3. (("delivery of health care"[MeSH Terms] OR "health services"[MeSH Terms] OR "dental health services"[MeSH Terms]
4. OR "health services accessibility"[MeSH Terms]
5. OR "services delivery system"[Title/Abstract] OR "health care service"[Title/Abstract]
6. OR "dental services"[Title/Abstract] OR "access to care"[Title/Abstract] OR "barriers"[Title/Abstract]
7. OR "health facilities"[Title/Abstract] OR hospital[Title/Abstract] OR "health center"[Title/Abstract]
8. OR "primary care"[Title/Abstract]))
9. AND
10. (("Southeast Asia"[MeSH Terms] OR "Southeast Asia"[Title/Abstract] OR SEA[Title/Abstract]
11. OR Thailand[Title/Abstract] OR Vietnam[Title/Abstract] OR Malaysia[Title/Abstract]
12. OR Indonesia[Title/Abstract] OR Philippines[Title/Abstract] OR Singapore[Title/Abstract]
13. OR Myanmar[Title/Abstract] OR Cambodia[Title/Abstract] OR Laos[Title/Abstract]
14. OR Brunei[Title/Abstract] OR "East Timor"[Title/Abstract] OR "Timor-Leste"[Title/Abstract]))

Filters: None applied (no restrictions on date, language, or study type).

**Scopus**

1. (("oral health" OR "dental health")
2. AND
3. ("services delivery system" OR "health care service" OR "dental services" OR "Access to care"
4. OR barriers OR "health facilities" OR hospital OR "health center" OR "primary care")
5. AND
6. ("Southeast Asia" OR SEA OR Thailand OR Vietnam OR Malaysia OR Indonesia OR Philippines
7. OR Singapore OR Myanmar OR Cambodia OR Laos OR Brunei OR "East Timor" OR "Timor-Leste"))

**Embase (via Ovid)**

1. ('oral health'/exp OR 'dental health'/exp OR 'oral health':ti,ab OR 'dental health':ti,ab)
2. AND
3. ('health care delivery'/exp OR 'health care service'/exp OR 'dental health service'/exp
4. OR 'health services accessibility'/exp
5. OR 'services delivery system':ti,ab OR 'health care service':ti,ab OR 'dental services':ti,ab
6. OR 'access to care':ti,ab OR barriers:ti,ab OR 'health facilities':ti,ab
7. OR hospital:ti,ab OR 'health center':ti,ab OR 'primary care':ti,ab)
8. AND
9. ('southeast asia'/exp OR 'southeast asia':ti,ab OR SEA:ti,ab OR Thailand:ti,ab
10. OR Vietnam:ti,ab OR Malaysia:ti,ab OR Indonesia:ti,ab OR Philippines:ti,ab
11. OR Singapore:ti,ab OR Myanmar:ti,ab OR Cambodia:ti,ab OR Laos:ti,ab OR Brunei:ti,ab
12. OR 'east timor':ti,ab OR 'timor-leste':ti,ab)

**Google Scholar** (first 200 most relevant results)

1. ("oral health" OR "dental health")
2. AND
3. ("services delivery system" OR "health care service" OR "dental services" OR "Access to care"
4. OR barriers OR "health facilities" OR hospital OR "health center" OR "primary care")
5. AND
6. ("Southeast Asia" OR SEA OR Thailand OR Vietnam OR Malaysia OR Indonesia OR Philippines
7. OR Singapore OR Myanmar OR Cambodia OR Laos OR Brunei OR "East Timor" OR "Timor-Leste")

### **WHO Global Burden of Disease (GBD) Database**

Search terms: oral health, dental health, Southeast Asia (Brunei, Cambodia, Indonesia, Laos, Malaysia, Myanmar, Philippines, Singapore, Thailand, Timor-Leste, Vietnam), service delivery, access, utilization, health system*.*

## Supplementary Table S1 Extracted service delivery domains from included studies

| Country | Author, Year | Comprehensiveness | Accessibility | Coverage | Continuity | Effectiveness | Safety | Patient-centeredness | Timeliness | Barriers |
| --- | --- | --- | --- | --- | --- | --- | --- | --- | --- | --- |
| Cambodia | Todd et al., 1994 |  | ✓ | ✓ |  | ✓ |  |  |  | ✓ |
|  | Chher et al., 2009 |  |  |  |  |  |  |  |  | ✓ |
|  | Turton et al., 2021 | ✓ | ✓ |  | ✓ | ✓ | ✓ | ✓ | ✓ |  |
| Indonesia | Pasiga et al., 2018 |  |  |  |  |  |  |  |  | ✓ |
|  | Akbar et al., 2019 |  | ✓ |  |  |  |  |  |  | ✓ |
|  | Dewanto et al., 2020 |  |  |  |  |  | ✓ |  |  |  |
|  | Santoso et al., 2020 |  |  |  |  | ✓ |  |  |  | ✓ |
|  | Akbar et al., 2022 |  | ✓ |  |  |  | ✓ |  |  | ✓ |
|  | Budi et al., 2023 | ✓ |  | ✓ |  |  | ✓ |  |  | ✓ |
|  | Khairinisa et al., 2024 | ✓ | ✓ |  |  |  |  |  |  | ✓ |
|  | Ramphoma et al., 2024 | ✓ |  |  | ✓ |  |  |  |  | ✓ |
| Malaysia | Jaafar et al., 1988 |  |  |  |  |  |  | ✓ |  |  |
|  | Jaafar et al., 1992 |  |  |  |  |  |  |  | ✓ |  |
|  | Chu et al., 2001 |  | ✓ |  |  |  |  |  |  |  |
|  | Othman et al., 2010 | ✓ | ✓ | ✓ | ✓ | ✓ |  |  |  |  |
|  | Saddki et al., 2010 |  |  |  |  | ✓ |  |  | ✓ | ✓ |
|  | AbuBakar et al., 2019 |  |  |  |  |  |  |  |  | ✓ |
|  | MdBohari et al., 2019 |  | ✓ | ✓ |  |  |  |  |  | ✓ |
|  | Blebil et al., 2020 |  |  |  |  |  | ✓ |  | ✓ | ✓ |
|  | Nurdin et al., 2020 |  |  |  |  | ✓ |  |  |  | ✓ |
|  | Benghasheer et al., 2022 |  |  |  |  |  |  |  |  | ✓ |
|  | AliNIM et al., 2023 |  | ✓ |  |  |  | ✓ |  |  | ✓ |
|  | Khairunjauhari et al., 2023 |  | ✓ |  |  |  |  | ✓ | ✓ | ✓ |
| Philippines | Ofilada et al., 2013 |  |  |  |  |  |  |  |  | ✓ |
| Singapore | Lo et al., 1991 |  | ✓ | ✓ | ✓ |  |  |  | ✓ | ✓ |
|  | Soh et al., 1991 | ✓ | ✓ | ✓ |  |  |  |  |  | ✓ |
|  | LimGXD et al., 2019 |  | ✓ |  |  |  |  |  |  | ✓ |
|  | Mittal et al., 2019 | ✓ | ✓ | ✓ | ✓ |  |  |  | ✓ | ✓ |
|  | TeoCHK et al., 2020 | ✓ | ✓ |  | ✓ | ✓ |  | ✓ | ✓ | ✓ |
| Country | Author, Year | Comprehensiveness | Accessibility | Coverage | Continuity | Effectiveness | Safety | Patient-centeredness | Timeliness | Barriers |
|  | Ho et al., 2023 |  |  |  |  |  |  |  |  | ✓ |
|  | Junnarkar et al., 2023 |  | ✓ |  |  |  |  |  | ✓ | ✓ |
|  | Ho et al., 2024 |  |  |  |  |  |  |  |  | ✓ |
|  | KohSMY et al., 2024 | ✓ |  |  |  |  |  |  |  | ✓ |
|  | TanSHX et al., 2024 |  |  | ✓ |  |  |  |  |  | ✓ |
| Thailand | Benjakul et al., 2000 | ✓ |  |  |  | ✓ |  |  |  | ✓ |
|  | Somkotra et al., 2009 |  | ✓ | ✓ |  |  |  |  |  |  |
|  | Somkotra et al., 2009 |  | ✓ | ✓ |  |  |  |  |  | ✓ |
|  | Tianviwat et al., 2009 | ✓ | ✓ | ✓ |  | ✓ |  |  |  |  |
|  | Tianviwat et al., 2009 | ✓ |  |  |  | ✓ |  |  |  | ✓ |
|  | Somkotra et al., 2013 |  | ✓ |  |  |  |  |  |  | ✓ |
|  | Somkotra et al., 2013 | ✓ | ✓ |  |  |  |  |  |  | ✓ |
|  | Chongthanavanit et al., 2018 |  |  |  |  |  |  |  |  |  |
|  | Sermsuti-Anuwat et al., 2018 |  | ✓ | ✓ |  |  |  | ✓ |  | ✓ |
|  | Limpuangthip et al., 2019 |  | ✓ |  |  |  |  |  |  | ✓ |
|  | Harirugsakul et al., 2020 |  | ✓ |  |  |  |  |  |  | ✓ |
|  | Thitasomakul et al., 2020 | ✓ | ✓ | ✓ |  | ✓ |  |  |  | ✓ |
|  | Chongthawonsatid et al., 2021 |  |  |  |  |  |  |  |  | ✓ |
|  | Somboonchan et al., 2022 | ✓ |  |  | ✓ | ✓ | ✓ | ✓ |  | ✓ |
|  | Chaianant et al., 2023 |  |  | ✓ |  |  |  |  |  |  |
| Vietnam | Dao et al., 2015 |  |  |  |  |  |  |  |  | ✓ |
| Brunei; Cambodia; Malaysia; Myanmar; Philippines; Indonesia; Singapore; Thailand; Vietnam | Petersen et al., 2012 | ✓ |  | ✓ |  |  |  |  |  | ✓ |
| Cambodia; Malaysia; Indonesia; Thailand | Ng et al., 2022 | ✓ |  | ✓ | ✓ | ✓ |  |  | ✓ | ✓ |
